# Malaria Prophylaxis Failure with Doxycycline, Central African Republic, 2014

**DOI:** 10.3201/eid2108.150524

**Published:** 2015-08

**Authors:** Marylin Madamet, Tiphaine Gaillard, Guillaume Velut, Cecile Ficko, Pascal Houzé, Claire Bylicki, Stéphane Mérat, Sandrine Houzé, Nicolas Taudon, Rémy Michel, Pierre Pasquier, Christophe Rapp, Bruno Pradines

**Affiliations:** Institut de Recherche Biomédicale des Armées, Brétigny sur Orge, France (M. Madamet, N. Taudon, B. Pradines);; Aix Marseille Université, Marseille, France (M. Madamet, N. Taudon, B. Pradines);; Centre National de Référence du Paludisme, Marseille (M. Madamet, N. Taudon, B. Pradines);; Hôpital d’Instruction des Armées Saint Anne, Toulon, France (T. Gaillard);; Centre d’Epidémiologie et de Santé Publique des Armées,; Marseille, France (G. Velut, R. Michel);; Hôpital d’Instruction des Armées Begin, Saint Mandé, France (C. Ficko, S. Mérat, P. Pasquier, C. Rapp);; Hôpital Saint-Louis, Paris, France (P. Houzé);; Hôpital Bichat Claude Bernard, Paris (S. Houzé);; Institut pour la Recherche et le Développement, Paris (S. Houzé),; Université Paris Descartes, Paris (S. Houzé);; Centre National de Référence du Paludisme, Paris (S. Houzé);; Antenne Médicale de Fontevraud, Fontevraud, France (C. Bylicki);; Ecole du Val-de-Grâce, Paris (R. Michel, C. Rapp)

**Keywords:** malaria, parasites, Plasmodium falciparum, doxycycline, Central African Republic, antimalarial drug, France, antimicrobial resistance, prophylaxis, travel, in vitro

**To the Editor:** Doxycycline is an effective antimalarial prophylactic drug when administered as a monotherapy 1 day before, daily during, and for 4 weeks after travel to an area where malaria is endemic (*1*). Doxycycline is currently a recommended chemoprophylactic regimen for travelers visiting areas where malaria is endemic and has a high prevalence of chloroquine or multidrug resistance (*2*). The World Health Organization also recommends doxycycline in combination with quinine or artesunate as the second-line treatment for uncomplicated *Plasmodium falciparum* malaria (*3*).

Prophylactic and clinical failures of doxycycline against *P. falciparum* have been associated with both inadequate doses (*4*) and poor patient compliance (*5*). However, resistance can also explain failures of prophylaxis. Cycline resistance in *Plasmodium* spp. has been documented as a consequence of selective drug pressure in a *P. berghei* murine malaria model (*6*). The administration of increasing doses of minocycline to mice infected with 1 × 10^7^ parasites for 86 successive passages over 600 days made it possible to obtain a resistant *P. berghei* strain with a median drug inhibitory concentration (IC_50_) of 600 mg/kg/d, which is 6-fold higher than that of the susceptible starting strain (100 mg/kg/d) (*6*). A Bayesian mixture modeling approach identified 3 different phenotypes (low, medium, and high doxycycline IC_50_ phenotypic groups) among *P. falciparum* clinical isolates (*7**,**8*). Using 90 isolates from 14 countries, we demonstrated that increases in copy numbers of *P. falciparum* metabolite drug transporter gene (*Pfmdt*, PFE0825w) and *P. falciparum* GTPase TetQ gene (*PfTetQ*, PFL1710c) are associated with reduced susceptibility to doxycycline (*9*); this association was later confirmed (*7*). In addition, isolates with *PfTetQ* KYNNNN motif repeats are associated with in vitro reduced susceptibility to doxycycline and with a significantly higher probability of having an IC_50_ above the doxycycline resistance threshold of 35 μM (*9**,**10*).

We report a case of documented malaria prophylactic failure with doxycycline in a 26-year-old soldier from France who was infected during a 6-week peacekeeping mission in the Central African Republic in 2014. According to his colleagues and the collective prophylaxis intake, the patient had been compliant with doxycycline prophylaxis. On admission to a hospital in Bangui, Central African Republic, the patient had fever (temperature 40°C), alteration of consciousness, and hypotension. The diagnosis of severe *P. falciparum* malaria was made on the basis of a rapid diagnostic test confirmed by a blood smear test (parasitemia 8% on day 0). Intravenous artesunate was immediately started, in accordance with World Health Organization recommendations (*3*). The patient’s clinical condition worsened, and kidney failure developed. Twenty-four hours later (day 1), he was transported by airplane to Bégin Military Teaching Hospital (Saint-Mandé, France). On admission, he had cerebral edema and a *P. falciparum* parasitemia level of 0.7%. The patient died 1 day later (day 2).

A blood sample obtained from the patient on day 1 in France showed a doxycycline concentration of 195 μg/mL plasma. This concentration, which was determined by liquid chromatography coupled with tandem mass spectrometry, was compatible with a last doxycycline uptake 1 day before diagnosis (day −1). The finding of the expected doxycycline plasma concentration, together with assurances (colleague’s statements and collective intake of doxycycline) that the patient had followed the drug regimen, was sufficient to suggest prophylaxis failure in a treatment-compliant patient. 

The *P. falciparum* sample obtained from the patient on arrival in France was evaluated for in vitro susceptibility to doxycycline, but the evaluation was unsuccessful. The number of copies of *PfTetQ* and *Pfmdt* genes were evaluated relative to the single-copy *P. falciparum β-tubulin* gene (*pfβtubulin*), as previously described (*7**,**8*). The sample was assayed in triplicate. The 2^–ΔΔCt^ method (where C*_t_* indicates cycle threshold) of relative quantification was used and adapted to estimate the number of copies of *Pfmdt* and *PfTetQ* by using the formula ΔΔC_t_ = (C_t (_*_PfTetQ_*_or_ *_Pfmdt_*_)_ − C_t (_*_Pfβtubulin_*_)_)_Sample_ – (C_t (_*_PfTetQ_*_or_ *_Pfmdt_*_)_ − C_t (_*_Pfβtubulin_*_)_)_Calibrator_. Genomic DNA extracted from 3D7 *P. falciparum*, which has a single copy of each gene, was used for calibrator sample; *Pfβtubulin* served as the control housekeeping gene. The experiment was assayed twice. The sample had 2 copies of *PfTetQ* and *Pfmdt* genes, which suggested decreased in vitro susceptibility of the sample to doxycycline (*8**,**9*). The genotyping of *PfTetQ* sequence polymorphisms was done by using conventional methods with the primers *PfTetQ* forward (5′-TCACGACAAATGTGCTAGATAC-3′) and *PfTetQ* reverse (5′-ATCATCATTTGTGGTGGATAT-3′), as previously described (*10*). Two *PfTetQ* KYNNNN motif repeats were found in the sample; <3 KYNNNN motif repeats are predictive of in vitro *P. falciparum*–resistant parasites with an IC_50_ of >35 μM (odds ratio 15) (*10*). The 2 copies of *Pfmdt* and the 2 KYNNNN motif repeats have been shown to be associated with parasites with in vitro resistance to doxycycline (*9**,**10*). The association of doxycycline resistance (prophylactic failure with statement of correct intake and the presence of an expected concentration) with increased *Pfmdt* copies and decreased *PfTetQ* KYNNNN motif repeats suggest that these molecular markers are predictive markers of doxycycline resistance that can be used for resistance surveillance.
